# Laminar Airflow in Penile Prosthesis Surgery and Infection: An Empty Systematic Review

**DOI:** 10.7759/cureus.99910

**Published:** 2025-12-23

**Authors:** Muhammad Umair Shafiq, Prasenjit Bhowmik, Sachin Yallappa, Mushtaq Hussain, Mustafa Ahmad

**Affiliations:** 1 Urology, University Hospitals Birmingham NHS Foundation Trust, Birmingham, GBR; 2 Urology, Queen Elizabeth Hospital Birmingham, Birmingham, GBR

**Keywords:** inflatable penile prosthesis, laminar airflow, operating room ventilation, penile prosthesis, prosthesis infection, prosthesis-related infections, surgical site infection, ultraclean ventilation, unidirectional airflow

## Abstract

Penile prosthesis (PP) infection is uncommon but serious. Laminar airflow/ultraclean ventilation (LAF/UCV) aims to reduce airborne bioburden, but its clinical impact on PP infection is unclear. We registered a protocol in PROSPERO (CRD420251128507) and followed PRISMA 2020/PRISMA-S, searching MEDLINE (PubMed), Embase (Ovid), Cochrane CENTRAL, CINAHL, and Dimensions (no date limits), screening reference lists/forward citations, and reviewing professional guidance. Eligible studies were randomised or non-randomised comparative PP studies reporting infection by LAF/UCV versus conventional ventilation; portable high-efficiency particulate air (HEPA)/UV recirculators were excluded a priori. Two reviewers independently screened the results; meta-analysis was prespecified if appropriate, otherwise a narrative synthesis was used. Of 99 records (98 databases; 1 other), 26 duplicates were removed; 72 titles/abstracts were screened; 2 full texts were assessed. As a result, no study met the inclusion criteria, and no meta-analysis was possible. Indirect evidence from orthopaedic, breast, vascular, and cardiac surgery shows no consistent reduction in deep surgical-site infection with LAF compared with conventional ventilation under real-world conditions where unidirectionality can be disrupted by door openings, staff/equipment movement, and thermal plumes. Evidence from other specialties is indirect and of uncertain applicability to penile prosthesis surgery. No eligible comparative penile prosthesis studies evaluating LAF/UCV versus conventional ventilation were identified; this is therefore an empty systematic review. Pending procedure-specific data, practice should prioritise multimodal infection-prevention bundles (e.g., antibiotic prophylaxis, "no-touch" technique, traffic control) and verification of ventilation performance during live use; future multicentre observational studies or cluster/stepped-wedge trials should prospectively capture ventilation parameters and key confounders to enable robust comparisons and cost-effectiveness analyses.

## Introduction and background

Penile prosthesis implantation is a durable and effective solution for refractory erectile dysfunction, with high patient satisfaction but a small risk of device infection that can lead to explantation, reoperation, and substantial cost, particularly in revision procedures and among patients with diabetes or immunosuppression [1,2]. Reported infection rates after penile prosthesis implantation are low in modern practice but remain clinically important. In a large statewide database of initial inflatable penile prosthesis (IPP) insertions, the overall infection rate was 2.3%, with infections occurring in 3% of men with diabetes versus 2% of those without diabetes [1]. A contemporary systematic review found that infection incidence was about 8-11% in early series, 3-5% in early 2000s cohorts, and approximately 0.3-2.7% in modern series using coated devices and refined surgical techniques; across these studies, reoperations consistently had higher infection rates than primary implants. Diabetes further increases infection risk, with a pooled odds ratio of 1.53 (95% CI: 1.15-2.04) for diabetic versus non-diabetic patients. Prosthesis infection, therefore, remains a feared complication because it leads to reoperation, loss of function, and increased healthcare costs [2].

However, because PP infection is generally considered predominantly contact-driven (skin/perineal flora and device handling), it remains unclear whether further reducing airborne bioburden with laminar airflow/ultraclean ventilation confers any measurable additional benefit. Penile prosthesis infection is generally considered an intraoperative inoculation event. In contrast to some other implant operations where airborne bioburden has been emphasised, the dominant concern in PP surgery is usually contact contamination from skin/perineal flora introduced to device components or sterile working surfaces during handling, followed by bacterial adherence and biofilm formation on the implant. Consequently, most established prevention strategies in PP implantation prioritise meticulous skin preparation and draping, strict device-handling discipline, and consistent perioperative antibiotic protocols.

PP implantation also has procedure-specific workflow features that influence contamination opportunities. In inflatable PP surgery (commonly via penoscrotal or infrapubic approach), prosthesis components are opened on adjacent sterile work surfaces and transferred repeatedly between the instrument table and the operative field during measurement, corporal dilation, cylinder insertion, pump placement, and reservoir placement. This "open-and-handle" workflow provides the main pathway for contact-driven contamination. Nevertheless, airborne bacteria-carrying particles could plausibly contribute indirectly by depositing onto exposed sterile work surfaces or device components during handling, and then being transferred to the implant. This is the biological rationale for why theatre ventilation performance and laminarity are sometimes considered in clean implant surgery.

Against this predominantly contact-driven infection pathway in PP surgery, the incremental clinical value of reducing airborne bioburden remains uncertain; however, laminar airflow/ultraclean ventilation (LAF/UCV) is still used in many "clean implant" theatres and therefore warrants procedure-specific evaluation. LAF/UCV systems aim to deliver unidirectional airflow with high air-exchange rates over the sterile field to reduce airborne bioburden; however, clinical data from high-volume arthroplasty and other clean implant surgeries have not shown a consistent reduction in deep surgical site infection (SSI) compared with conventional ventilation [3-6]. For example, cross-specialty syntheses have not demonstrated consistent reductions in SSI with LAF compared with conventional ventilation, underscoring the need for PP-specific comparative evidence. Performance is sensitive to real-world factors like door openings, staff movement, heat plumes, and field crowding that disrupt unidirectionality (laminarity) and may dilute any clinical effect [7,8]. Heterogeneity in system design, commissioning, maintenance, and local practice further drives variability; reductions in airborne particles may be necessary but are not sufficient to lower SSI risk. Preventive strategies centre on evidence-based perioperative bundles (antibiotic prophylaxis, skin preparation, draping, glove and instrument protocols) [9,10] alongside device innovations (antibiotic-impregnated coatings, hydrophilic sleeves) and operating theatre environmental controls, including ventilation design [11,12]. In this review, the 'operative field' refers to the patient's surgical site and the immediately adjacent sterile working surfaces (e.g., Mayo stand and back/instrument tables) where prosthesis components and instruments are opened and handled.

Penile prosthesis implantation is a clean implant procedure, typically performed without entry into the urinary tract, and therefore shares some conceptual features with other prosthetic surgeries. However, the operative field, extent of exposure, and workflow differ from larger-joint or high-exposure implant operations, which may limit direct extrapolation of ventilation-related findings. Infection risk in PP surgery is also strongly shaped by patient and procedural factors such as diabetes, revision surgery, and prolonged operative time, which are consistently associated with increased penile implant infection risk.

The central unresolved problem is that, despite the use and cost implications of LAF/UCV in clean implant theatres, there is no penile prosthesis-specific comparative clinical evidence evaluating LAF/UCV versus conventional ventilation for prosthesis infection outcomes. Cross-specialty findings are mixed and of uncertain transferability to penile prosthesis implantation due to differences in procedural exposure, workflow, and baseline risk. Thus, the clinical and economic justification for LAF/UCV in penile prosthesis surgery remains unresolved, warranting a focused systematic review.

Given the cost and implementation implications of LAF/UCV, a systematic review was warranted to determine whether procedure-specific comparative evidence exists; identifying an evidence gap is itself informative for research prioritisation. Ventilation performance depends on system design (diffuser geometry, supply velocity) and in-use room behaviour; implant procedures differ in exposure, duration, and workflow, so extrapolating orthopaedic findings to penile prosthesis surgery is uncertain. We distinguish ceiling-mounted unidirectional flow (ultraclean) canopies (LAF/UCV) from portable high-efficiency particulate air (HEPA)/UV recirculators, which recirculate room air and are not considered LAF/UCV operating theatre ventilation under engineering/standards guidance [11-13].

We also reviewed cross-specialty evidence on laminar airflow and infection rates, including high-volume arthroplasty, implant-based breast reconstruction [14], prosthetic vascular grafting [15], and cardiac surgery [16] to assess applicability to penile prosthesis surgery. These indirect data are considered contextual and appraised further in the discussion section. Accordingly, any cross-specialty data are presented here as contextual background only and should not be interpreted as procedure-specific effectiveness evidence.

Baseline infection rates in penile prosthesis surgery are low: a contemporary review places modern primary inflatable penile prosthesis (IPP) infection rates at approximately 1% in typical series [17]. In a large 'no-touch' cohort, primary implantation infection rates were below 1%, markedly lower than historical practice [18]. A recent overview likewise estimates approximately 1% for primary implants, with higher rates in revisions and among patients with diabetes or immunosuppression [19]. These uncertainties mirror broader operating room ventilation literature, which emphasises that ventilation system type, diffuser geometry, and in-use theatre behaviour jointly determine airflow patterns and contamination risk [20]. Accordingly, this empty systematic review evaluates whether any penile prosthesis-specific comparative clinical evidence exists for LAF/UCV versus conventional ventilation and, in its absence, delineates a targeted research and reporting agenda for decision-grade future studies.

## Review

Methodology

Protocol and Registration

The protocol was registered in PROSPERO (CRD420251128507). Reporting followed PRISMA 2020; complete search strategies are documented per PRISMA-S. Meta-analysis was prespecified if sufficient comparable studies were identified; otherwise, a structured narrative synthesis was planned in accordance with synthesis without meta-analysis (SWiM) guidance. After piloting, we removed planned date limits and re-ran searches without date restrictions; this change has been recorded as an amendment in PROSPERO. The results presented reflect the no-date-limit strategies in Appendix 1.

Eligibility Criteria

Eligible studies were randomised or non-randomised comparative penile prosthesis (PP) studies enrolling adults (≥18 years) undergoing inflatable or malleable PP implantation (primary or revision) and reporting postoperative infection outcomes stratified by ceiling-mounted LAF/UCV versus conventional operating-room ventilation. We focused on fixed theatre ventilation systems (LAF/UCV canopies/engineered room systems); portable HEPA/UV or other mobile air-cleaning/airflow devices (e.g., portable recirculators or mobile LAF units) were excluded a priori as adjunct interventions outside the prespecified scope. Studies of other surgical specialties and non-clinical engineering/computational fluid dynamics (CFD)** **or airflow-only studies (without patient infection outcomes) were not eligible and are cited only as contextual background.

The exposure was ceiling-mounted unidirectional airflow delivered by an ultraclean/laminar airflow canopy (LAF/UCV). The comparator was conventional (mixing/turbulent) operating-theatre ventilation without LAF/UCV. The primary outcome was PP infection as defined by individual studies (including infection requiring reoperation, such as explantation or revision, where reported). Secondary outcomes were any surgical site infection (superficial, deep, or organ/space), time to infection (≤90 vs >90 days), revision-free survival, and microbiology. We did not apply a minimum postoperative follow-up threshold; studies with any reported follow-up duration were eligible. Where available, we planned to extract follow-up length and categorise infection timing (≤90 vs >90 days) as prespecified. Co-interventions (e.g., antibiotic coatings, "no-touch" technique, perioperative antibiotics) and key comorbidities were extracted as potential confounders/effect modifiers where reported.

Information Sources

We searched MEDLINE (PubMed), Embase (Ovid), Cochrane CENTRAL, CINAHL, and Dimensions, and screened reference lists and forward citations. We also reviewed professional guideline websites (American Urological Association, AUA, and European Association of Urology, EAU) for guidance related to penile prosthesis surgery and perioperative infection prevention. Databases and registers were searched on 27-28 September 2025 (ClinicalTrials.gov, WHO International Clinical Trials Registry Platform, ICTRP, ISRCTN); guideline websites were checked on October 5, 2025. Full verbatim strategies are provided in Appendix 1 (PRISMA-S).

Search Strategy

The search was structured around three concepts aligned to the review objective: (1) penile prosthesis implantation, (2) operating-room ventilation mode (LAF/UCV versus conventional/mixing), and (3) surgical-site/device infection. We searched MEDLINE (PubMed), Embase (Ovid), Cochrane CENTRAL, CINAHL, and Dimensions from inception to September 28, 2025; the final database search was run on September 28, 2025, with an English-language limit applied. Searches combined controlled vocabulary and keywords for penile prosthesis, operating-theatre ventilation, and laminar/unidirectional flow, and infection outcomes. Engineering/CFD terminology was not required for eligibility and was therefore confined to a supplementary search block (term set) appended to the ventilation concept to capture potentially misindexed/variably indexed ventilation reports; all retrieved records were still screened against the prespecified clinical eligibility criteria for comparative PP studies. Full search strategies for all databases, registers, and websites, copied exactly as run (including field tags, Boolean/proximity operators, and all limits/filters and run dates), are provided in Appendix 1 in accordance with PRISMA 2020 and PRISMA-S/PRISMA-Search.

Selection Process

Records were de-duplicated in EndNote and imported into Rayyan for screening management. Two reviewers independently screened titles/abstracts, and then full-text reports were retrieved for all records deemed potentially eligible by either reviewer. Disagreements were resolved by discussion and consensus, with third-reviewer adjudication when required. No records were excluded based solely on automation; Rayyan was used as a screening platform rather than as an automated decision tool. Reasons for full-text exclusion were recorded prospectively and are listed in Appendix 2. 

Data Collection Process

We prespecified and piloted a data-extraction form capturing study and participant characteristics; ventilation variables (presence and type of LAF/UCV canopy, canopy/diffuser geometry, air-change rate, supply air velocities, and any in-use verification such as smoke visualisation or anemometry); co-interventions; outcomes; follow-up time points; and analysis set. Methods followed standard guidance. No eligible comparative studies were included; therefore, no data extraction was undertaken.

Data Items and Outcomes

Data items were defined a priori. Outcomes of interest were prosthesis infection requiring reoperation (primary) and the secondary outcomes listed above. Where reported, time-to-event metrics and microbiology were to be extracted alongside the analysis set used.

Risk of Bias Assessment

Risk of bias assessment was prespecified for any eligible comparative studies. Randomised trials would have been assessed using the Cochrane Risk of Bias 2 (RoB 2) tool, and non-randomised comparative studies using Risk of Bias in Non-randomized Studies - of Interventions (ROBINS-I). Two reviewers would have assessed risk of bias independently, with disagreements resolved by consensus or third-reviewer adjudication, and an overall judgement (low/some concerns/high for RoB 2; low/moderate/serious/critical for ROBINS-I) derived according to tool guidance. Because no eligible comparative penile prosthesis studies were identified, no risk of bias assessments were performed.

Effect Measures

For dichotomous outcomes, we planned risk ratios (RRs); for continuous outcomes, mean difference (MD) or standardised mean difference (SMD), each with 95% confidence intervals; and for time-to-event outcomes, hazard ratios (HRs), where reported. As no eligible comparative studies were included, no effect measures were calculated.

Synthesis Methods

We prespecified a random-effects meta-analysis when two or more clinically and methodologically comparable studies reported the primary outcome. For dichotomous outcomes, we planned to synthesise effect estimates as risk ratios (RR) with 95% confidence intervals; time-to-event outcomes (if reported) would be summarised using hazard ratios. Between-study heterogeneity would be assessed using I² and τ² and explored using prespecified subgroup analyses (primary versus revision surgery, device type, diabetes status, antibiotic coating, and ventilation parameters). Analyses would have been conducted using standard meta-analysis software (e.g., RevMan or R). If ≥10 studies had contributed to a meta-analysis, we planned to assess small-study effects visually (funnel plot) and, where appropriate, with statistical tests; otherwise, reporting-bias assessment would not be informative. If meta-analysis was not appropriate, we planned a structured narrative synthesis with transparent grouping and reporting consistent with SWiM guidance. Because no eligible comparative penile prosthesis studies were identified, no synthesis of intervention effects was possible, and results are limited to a descriptive summary of the search and study-selection process. The discussion, therefore, focuses on the interpretation of the evidence gap; any cross-specialty material is presented explicitly as indirect contextual background and not as evidence of PP effectiveness.

Certainty of Evidence

We planned to rate certainty for key outcomes using the Grading of Recommendations Assessment, Development and Evaluation (GRADE) approachif comparable effect estimates became available; with no eligible comparative studies, a GRADE assessment was not performed.

Ventilation Definitions

We adopted engineering/standards-based definitions of LAF/UCV: a ceiling-mounted unidirectional canopy delivering uniform, low-turbulence flow across a protected zone, validated at design and subject to routine testing. This is distinct from portable recirculating devices, which do not establish a canopy flow field and therefore do not constitute LAF/UCV.

Results

Study Selection

**Figure 1:**
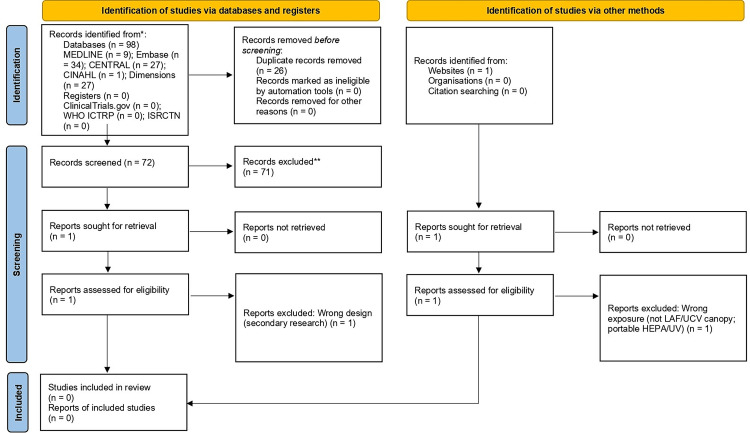
PRISMA 2020 flow diagram - study selection for LAF/UCV versus conventional ventilation in penile prosthesis surgery Records identified: databases n=98 (MEDLINE 9; Embase 34; CENTRAL 27; CINAHL 1; Dimensions 27); other methods n=1; registers n=0. Duplicates removed n=26; screened n=72; reports sought and assessed for eligibility n=2; not retrieved n=0; exclusions n=2 (wrong design; wrong exposure - portable HEPA/UV); included n=0. LAF - laminar airflow; UCV - ultraclean ventilation; HEPA - high-efficiency particulate air; UV - ultraviolet; ICTRP - International Clinical Trials Registry Platform; ISRCTN - International Standard Randomised Controlled Trial Number Created by authors. Image Credit: Muhammad Umair Shafiq

Searches identified 99 records (databases n=98; registers n=0; other sources n=1). After removing 26 duplicates, 72 titles/abstracts were screened, and 71 were excluded. Two reports were assessed in full - Mulcahy 2014 (review) [21] and a HUAIRS meeting abstract (portable HEPA/UV) [22] - and both were excluded (no LAF/UCV canopy comparison with infection outcomes). No comparative penile prosthesis studies stratifying infection outcomes by LAF/UCV versus conventional ventilation were included; therefore, no meta-analysis or other quantitative synthesis was performed. Study selection is shown in Figure 1 (PRISMA 2020 flow), and full-text reports assessed and excluded with primary reasons are summarised in Appendix 2. Detailed PRISMA 2020 node-by-node counts are provided in Appendix 3. 

Study Characteristics

No eligible comparative penile prosthesis studies were included; accordingly, a table of included study characteristics is not presented.

Risk of Bias

Not applicable, as no comparative penile prosthesis studies met the inclusion criteria and no risk-of-bias assessment could be performed.

Synthesis of Results

No effect estimates, 95% confidence intervals, heterogeneity assessments (e.g., I²), subgroup analyses, or sensitivity analyses were possible because no eligible comparative studies were identified. Meta-analysis and meta-regression were therefore infeasible, and no structured synthesis of comparative effects could be undertaken. Risk-of-bias assessment and certainty of evidence (e.g., GRADE) were not performed for the same reason. Accordingly, this empty systematic review is limited to documenting the absence of penile prosthesis-specific LAF/UCV comparative evidence. 

Discussion

Principal Finding and Evidence Gap

This is an empty systematic review: despite comprehensive searches across databases, trial registries, and citation pathways, no eligible comparative penile prosthesis studies assessed ceiling-mounted LAF/UCV versus conventional operating-room ventilation for infection outcomes. As a result, PP-specific effectiveness of LAF/UCV cannot be determined from current evidence, and this review cannot adjudicate the relative importance of airborne versus contact-driven pathways in PP infection. The review focuses on fixed theatre ventilation systems and does not address portable adjunct air-cleaning or mobile airflow devices; generalisability to these technologies is therefore limited. The primary contribution is to document a procedure-specific evidence gap and to outline the minimum data elements required for decision-grade future studies. Baseline infection rates after primary IPP are low, even at academic training centres, which implies that trials powered for infection endpoints would require very large samples [17-19]. Evidence is currently insufficient to recommend for or against ceiling-mounted LAF/UCV for penile prosthesis infection prevention. Table 1 summarises typical infection rates for penile prosthesis and other implant-related surgeries, providing quantitative context for baseline risk.

**Table 1:** Typical infection rates in implant-related surgery This table provides approximate infection rates across penile prosthesis and other implant-related surgeries to contextualise baseline risk. Data are drawn from arthroplasty registries/meta-analyses [3-6], an implant-based breast reconstruction cohort [14], vascular graft surgery reports [15], and cardiac surgery analyses [23].

Surgery type	Approximate infection rate (surgical site infection)	References
Penile prosthesis (primary implant)	≈1% (≈10 per 1,000)	[17-19]
Penile prosthesis (revisions/high-risk)	≈3-5% (higher with diabetes/revision)	[1,2,19]
Hip/knee arthroplasty (primary)	≈1-2%	[3-6]
Cardiac surgery (sternotomy; all sternal wound infections)	≈6-7% at 90 days (prospective cohort)	[23]
Vascular graft surgery	Variable by site; often <5%	[15]
Breast implant reconstruction	≈2-24% (higher in high-risk cases)	[14]

Implications for Penile Prosthesis Practice: Mechanistic Rationale, Real-World Performance, and Generalisability

Penile prosthesis infection appears predominantly contact-driven, linked to skin and operative handling, as reflected by the predominance of skin flora in cultures and the marked reductions in infection with antibiotic-coated devices and "no-touch" techniques. Nevertheless, airborne deposition on implants remains biologically plausible and provides a theoretical rationale for LAF/UCV, even though evidence from other implant specialties has not shown consistent clinical benefit over conventional ventilation.

Laminar airflow is intended to deliver unidirectional, low-turbulence, HEPA-filtered supply air across a protected zone so that contaminants are swept away from the incision and instrument field under controlled conditions [11,12]. In practice, real-world theatre behaviour and door openings, staff and equipment movement, instrument tables placed within the canopy, and thermal plumes from lights and warming devices can disturb unidirectionality and create mixing, recirculation, or dead zones, so that LAF performance becomes highly implementation-sensitive [7,8,11,12,20]. Key LAF/UCV perturbations, mechanistic airflow effects, and practical mitigations are summarised in Appendix 4.

**Figure 2:**
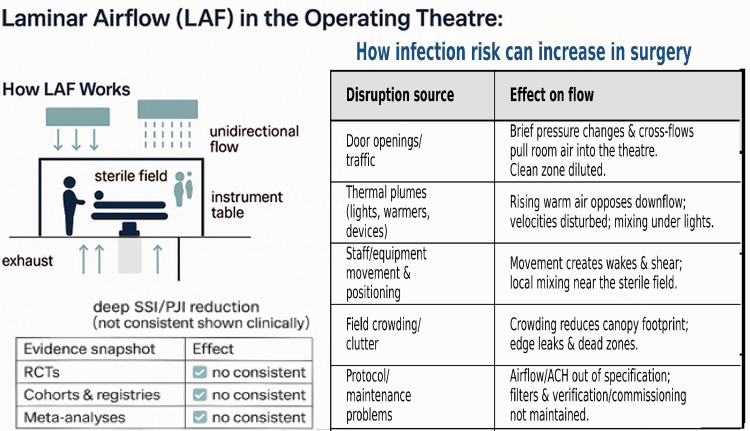
Laminar airflow: ideal unidirectional downflow and common disruption pathways in the operating theatre Left: ceiling-mounted UCV canopy supplying uniform, low-turbulence downflow over the sterile field with low-level peripheral returns. Right: common disruptions that break laminarity - door openings/traffic, thermal plumes from lights/warmers/devices, staff/equipment movement and positioning, field crowding/clutter, and protocol/maintenance problems - leading to mixing, dead zones and recirculation. The evidence snapshot shows that deep SSI/PJI reduction with LAF has not been consistently demonstrated in clinical studies versus conventional ventilation, underscoring the need for robust in-use performance and workflow discipline. LAF - laminar airflow; UCV - ultraclean ventilation; SSI - surgical-site infection; PJI - prosthetic joint infection; ACH - air changes per hour. Created by authors. Image Credit: Muhammad Umair Shafiq

Penile prosthesis implantation is a clean implant procedure, typically performed without entry into the urinary tract, and therefore shares some conceptual features with other prosthetic surgeries. However, the operative field, extent of exposure, and workflow differ from larger-joint or high-exposure implant operations, which may limit direct extrapolation of ventilation-related findings. Infection risk in PP surgery is also strongly shaped by patient and procedural factors such as diabetes, revision surgery, and prolonged operative time, which are consistently associated with increased penile implant infection risk. In the absence of PP comparative data, cross-specialty LAF results should therefore be interpreted as indirect, hypothesis-generating context rather than as evidence to support or refute LAF for penile prosthesis infection prevention. The excluded HUAIRS report [22] evaluated a portable HEPA/UV room-air recirculation unit rather than a ceiling-mounted LAF/UCV canopy and did not report comparative penile prosthesis infection outcomes, so it did not meet our inclusion criteria [11-13]. Figure 2 illustrates the intended unidirectional downflow pattern and the common intra-operative perturbations that can degrade laminarity and create mixing or recirculation.

Contextual (Indirect) Evidence From Other Specialties

Table 2 summarises indirect evidence on LAF/UCV and infection outcomes across other surgical fields. The following evidence is presented for contextual background only and should not be interpreted as penile prosthesis-specific comparative effectiveness evidence. The distribution and overall direction of clinical findings across specialties are summarised in Appendix 5.

**Table 2:** Indirect evidence on LAF and infection outcomes in other surgical specialties These findings are presented as indirect contextual evidence and are of uncertain applicability to penile prosthesis implantation; they do not constitute PP-specific comparative effectiveness data. Data are drawn from arthroplasty registries/meta-analyses [3-6], an implant-based breast reconstruction cohort [14], vascular graft surgery reports [15], and cardiac surgery analyses [16]. LAF - laminar airflow; UCV - ultraclean ventilation; PJI - prosthetic joint infection

Surgical field	Summary of LAF effect on infection rates	References
Orthopaedic (hip/knee arthroplasty)	Large registries and meta-analyses show no meaningful reduction in deep PJI with LAF; overall findings are inconsistent/neutral.	[3-6]
Breast (implant-based reconstruction)	No benefit of LAF versus conventional ventilation in the available comparative cohort.	[14]
Vascular (prosthetic graft surgery)	No clear reduction in graft/patch infection with LAF; evidence is limited, heterogeneous, and confounded.	[15]
Cardiac surgery (sternotomy)	Mixed findings; after adjustment, several analyses show no significant difference in sternal wound infection.	[16]

Overview (surrogates vs outcomes): Bench and in-room testing consistently shows fewer airborne particles and colonies under laminar systems in controlled conditions [11,12]. However, across registries and pooled analyses in hip and knee arthroplasty, any reduction in prosthetic joint infection (PJI) with LAF is inconsistent, and some studies paradoxically report higher infection rates in LAF theatres [3-6].

These observations likely reflect the sensitivity of laminar systems to real-world perturbations (door openings, staff/equipment movement and positioning, thermal plumes) that break unidirectionality and draw room air into the field [7,8]. Heterogeneity in system design, commissioning, maintenance, and behaviour-dependent performance may further contribute [11,12].

Orthopaedic context (hip/knee arthroplasty): Data spanning randomised comparisons, large cohorts, and multiple meta-analyses generally do not show a clinically meaningful PJI reduction with LAF versus conventional ventilation; when advantages are reported, they tend to be small and implementation-sensitive [3-6]. The orthopaedic experience underscores that environmental engineering gains do not automatically translate to outcome gains without disciplined workflow and verified performance in use.

Vascular context: Evidence specific to open vascular procedures with prosthetic material is sparse, methodologically heterogeneous, and often limited by incomplete reporting of ventilation variables. The few comparative analyses do not demonstrate a clear reduction in graft/patch infection with laminar airflow (LAF), and confounding from bundled co-interventions (antibiotic protocols, operating theatre (OT) traffic policies including door-discipline and personnel-movement controls) remains a concern [15].

Cardiac context: Comparative studies assessing LAF and sternal wound infection are few; effects are mixed and often attenuate after adjustment for confounders [16]. Ventilation descriptors are inconsistently reported [16], and any air-quality improvements can be diluted by door discipline and traffic patterns that degrade laminarity during active cases [7,8]. Sternal surgical wound infection is uncommon but clinically significant after cardiac surgery (prospective n=1,004; 90-day sternal wound infection = 6.8%), which helps explain why airflow choices are periodically evaluated in that field [23].

Overall, cross-specialty outcomes should be treated as an indirect, implementation-sensitive context, not procedure-specific evidence for or against LAF/UCV in penile prosthesis infection prevention.

Implementation, Bias, and Resource Considerations

Because LAF/UCV performance in practice is highly workflow-dependent, any interpretation should consider in-use performance rather than commissioning alone; simple checks (e.g., smoke/fog visualisation and hot-wire or vane anemometry) can help confirm canopy alignment and centre/edge velocities and identify cross-drafts or recirculation that static certification may miss [11,12]. Where available, particle counting and settle plates provide complementary but imperfect surrogates for deep SSI risk, so environmental optimisation should be interpreted alongside routine infection surveillance rather than in isolation [24]. Portable HEPA units and mobile "mini-LAF" canopies may improve local air cleanliness when the primary canopy is small, misaligned, or frequently perturbed, but outcome-level evidence that these adjuncts reduce penile prosthesis infection remains scant and low-certainty [13,19]; any deployment should be framed as an engineering control within a broader infection-prevention bundle and evaluated with both environmental surrogates and patient outcomes using adequate follow-up [16-18,25]. Across non-randomised comparisons, allocation to LAF theatres is rarely random and is vulnerable to confounding by case-mix, bundled co-interventions, and exposure misclassification (when "LAF rooms" do not consistently deliver unidirectionality during live cases) [7,8,11,12]. Key bias pathways and design safeguards are summarised in Appendix 6.Finally, given the substantial capital, energy, and maintenance demands of LAF/UCV, opportunity costs and sustainability should be weighed against alternative investments (e.g., training, surveillance infrastructure, device/process improvements), while lower-cost portable HEPA/UV adjuncts merit economic modelling but any cost-effectiveness claims still depend on demonstrating clinical benefit, which currently remains unproven [11-13,16,19,25]. 

Limitations

This empty systematic review is primarily limited by indirectness: in the absence of penile prosthesis-specific comparative data, interpretation necessarily relies on cross-specialty ventilation literature whose applicability is constrained by differences in operative field, exposure, workflows, and baseline risk. Consequently, the review can document an evidence gap but cannot estimate the effect of LAF/UCV on penile prosthesis infection outcomes.

Regarding review process and scope limitations, searches were restricted to English-language records, which may have reduced retrieval sensitivity and may have missed relevant non-English evidence (language bias). Additionally, ventilation mode is inconsistently described and indexed in surgical studies; therefore, relevant penile prosthesis studies may be difficult to retrieve if theatre ventilation mode is not explicitly reported, contributing to an apparent evidence gap. In addition, we prespecified the exposure as ceiling-mounted LAF/UCV systems and excluded portable/recirculating adjunct air-cleaning devices; therefore, the findings should be interpreted as "no eligible comparative evidence identified" for ceiling-mounted systems and cannot inform the incremental benefit of adjunct portable technologies. These constraints should not be interpreted as evidence of no effect. To contextualise current guidance on LAF/UCV and SSI prevention, Table 3 summarises positions from major international bodies and relevant standards documents.

**Table 3:** Guidelines and standards on LAF for SSI prevention: summary of positions Positions of major bodies on LAF and SSI: WHO issues a conditional recommendation against using laminar airflow to reduce SSI in total arthroplasty [9]; CDC/HICPAC offers no recommendation (unresolved issue) on performing orthopaedic implant operations in LAF rooms [10]; ANSI/ASHRAE/ASHE Standard 170 specifies ventilation design/performance requirements (e.g., ACH, pressure relationships, filtration, temperature/humidity) rather than clinical-outcomes guidance [11]; UK HTM 03-01 sets design, commissioning, verification, and operational-management requirements for specialised ventilation (engineering/operational guidance, not outcomes) [12]. LAF - laminar airflow; SSI - surgical-site infection; ACH - air changes per hour; OR/OT - operating room/theatre; CDC/HICPAC - Centers for Disease Control and Prevention/Healthcare Infection Control Practices Advisory Committee; ASHRAE/ASHE - American Society of Heating, Refrigerating and Air-Conditioning Engineers/American Society for Health Care Engineering; HTM - Health Technical Memorandum

Body/document	Position on LAF and SSI	Scope/emphasis	References
WHO (2016/2018)	Advises against using LAF to reduce SSI in total arthroplasty (conditional recommendation against).	Prioritises SSI-prevention bundles; general ventilation/traffic discipline; no LAF endorsement.	[9]
CDC/HICPAC (2017)	No endorsement of LAF for SSI prevention (unresolved); no recommendation to use LAF.	Emphasises standard OR/theatre ventilation (positive pressure, ACH) and minimising door openings.	[10]
ANSI/ASHRAE/ASHE Standard 170	Sets ventilation performance requirements (ACH, filtration, pressure, temperature).	Engineering/standards document; not clinical outcomes evidence.	[11]
UK HTM 03-01	Defines design, commissioning, verification and maintenance requirements.	Engineering/operational performance; in-use verification; not outcomes guidance.	[12]

Practice and Research Implications

In the absence of penile prosthesis-specific comparative evidence, it is reasonable for practice and local policy to prioritise established multicomponent infection-prevention bundles (e.g., coated devices, standardised prophylaxis, meticulous skin antisepsis, and "no-touch" technique), behavioural measures (door discipline, equipment zoning), and in-use ventilation performance verification over binary mandates about the presence or absence of LAF/UCV. Current international positions are broadly consistent with this approach: WHO SSI guidance advises against using LAF specifically to reduce SSI in primary total arthroplasty, and CDC guidance emphasises meeting standard operating theatre ventilation requirements and minimising traffic rather than endorsing LAF for SSI reduction [9,10]. Where decommissioning or replacement of LAF systems is contemplated, evidence-generating approaches (e.g., stepped-wedge implementation or interrupted time-series evaluation) could help ensure that local decisions yield generalisable knowledge beyond a single centre. For research, the key implication of this empty review is the need for procedure-specific comparative evidence. A pragmatic research agenda to generate decision-grade evidence is outlined in Appendix 7. Given low primary infection rates for penile prosthesis (approximately 1% in primary implants, higher in revisions and high-risk cohorts), individually randomised trials powered for infection endpoints would require very large samples [1,2,17-19]. Pragmatic alternatives include cluster-randomised or stepped-wedge designs that randomise at the theatre or hospital level and use routine registry capture for outcomes to improve feasibility and external validity. Observational approaches should build multicentre registries that systematically capture ventilation variables and relevant confounders (diabetes, revision status, operative duration/complexity, surgeon volume) [3-6,11-12]. Finally, cost-effectiveness analyses comparing infrastructure investments (LAF/UCV versus out-of-scope portable HEPA/UV adjuncts versus workflow optimisation) with plausible clinical benefits should accompany implementation efforts, recognising that economic conclusions depend on demonstrating clinical benefit, which currently remains unproven [13,16]. Appendix 8 summarises standard operating-theatre ventilation requirements and their links to infection prevention. Table 4 summarises key practice and research priorities to generate decision-grade evidence.

**Table 4:** Recommendations for practice and research LAF - laminar airflow; PP - penile prosthesis; OR - operating room; HEPA - high-efficiency particulate air; ACH - air changes per hour

For clinical practice (current management)	For future research (evidence gaps and priorities)
Prioritise prevention bundles (antibiotics, antisepsis, "no-touch", traffic control).	Embed ventilation variables (LAF status, canopy footprint, velocity targets, ACH, HEPA, door counts).
Maintain standard OR ventilation (positive pressure; doors closed).	Use robust designs: multicentre propensity; cluster/stepped-wedge randomisation.
Verify in-use laminarity (smoke; anemometry); remediate promptly.	Harmonise infection definitions; follow-up at 30/90/180/365 days.
Focus on environmental basics; document maintenance.	Evaluate cost-effectiveness and sustainability (LAF vs portable HEPA vs workflow).
Document each case (ventilation mode, door openings, occupancy).	Pre-register; report co-interventions and null results transparently.

## Conclusions

This systematic review identified no eligible comparative clinical studies evaluating ceiling-mounted LAF/UCV versus conventional operating-room ventilation for penile prosthesis infection outcomes. Consequently, current evidence is insufficient to recommend for or against LAF/UCV as a penile prosthesis-specific infection-prevention strategy.

In the interim, practice should prioritise established multicomponent infection-prevention bundles and disciplined intra-operative workflow. Future decision-grade evidence will require multicentre comparative designs with standardised reporting of ventilation parameters, co-interventions, and follow-up, alongside robust adjustment for case-mix and revision status.

## Figures and Tables

**Figure 3:**
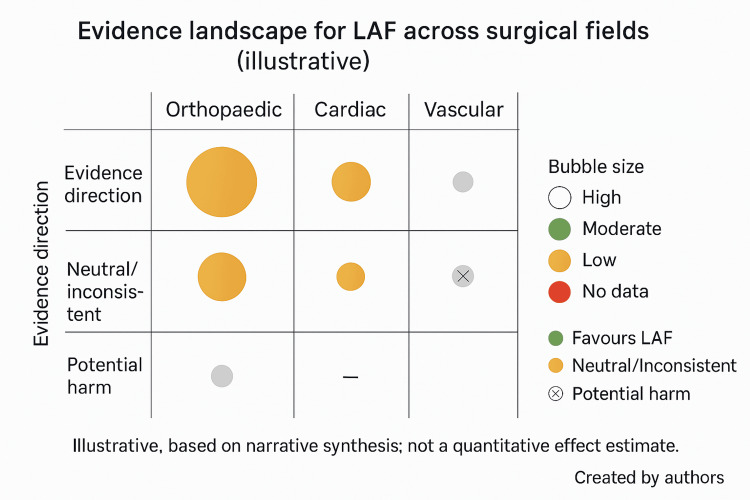
Contextual laminar-airflow evidence map by surgical specialty (illustrative) Bubble map summarising the narrative evidence on laminar airflow (LAF) by surgical field. Bubble size qualitatively reflects evidence volume (high, moderate, low); blank/dash cells indicate no data. Colour/symbol encodes direction: green - favours LAF; yellow - neutral/inconsistent; “×” - potential harm. Illustrative synthesis only; not a quantitative effect estimate. This map does not imply transferability to penile prosthesis surgery. LAF - laminar airflow Created by authors. Image Credit: Muhammad Umair Shafiq

**Figure 4:**
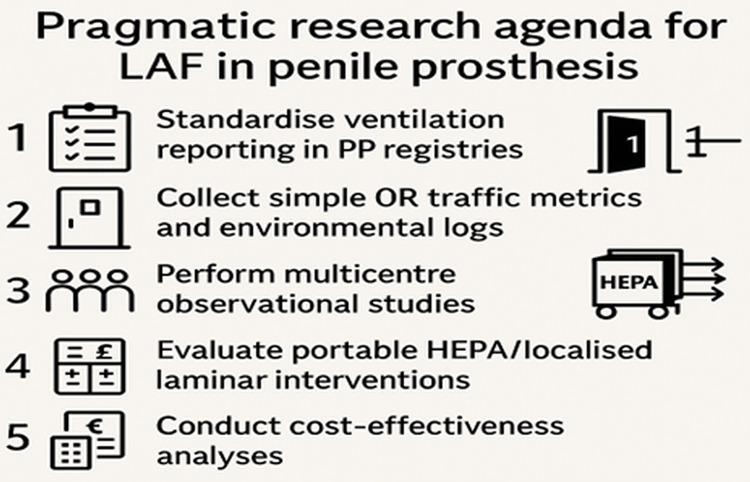
Research priorities to evaluate LAF in penile-prosthesis surgery Proposed priorities to generate decision-grade evidence on LAF for penile-prosthesis surgery: (1) standardise ventilation fields captured in PP registries (e.g., presence/type of canopy, ACH, pressure, in-use verification); (2) collect simple operating-room (OR) traffic metrics and environmental logs (e.g., door cycles, staff counts), given links between traffic and contamination; (3) run multicentre observational studies with preregistered analysis plans and risk-of-bias control aligned with STROBE/ROBINS-I; (4) pragmatically evaluate portable HEPA or localised unidirectional-flow adjuncts in real ORs (pragmatic/cluster/stepped-wedge designs as appropriate); (5) conduct cost-effectiveness analyses following CHEERS 2022. Evidence for LAF's clinical benefit remains inconsistent in orthopaedics, underscoring the need for focused, context-specific evaluation. LAF - laminar airflow; PP - penile prosthesis; OR - operating room; HEPA - high-efficiency particulate air (filter); ACH - air changes per hour. Created by authors. Image Credit: Muhammad Umair Shafiq

## Appendices

Appendix 1: PRISMA-S - information sources and verbatim strategies

Scope/limits

All databases and registries were last searched 27-28 September 2025; no date limits; English only (resource constraints; most PP literature indexed in English). Guideline websites were checked on 5 October 2025. Strategies are reported verbatim (field tags, Boolean/proximity operators, limits, and dates preserved).

Databases

*MEDLINE (via PubMed)* - searched 27-28 September 2025; field tags used to disable ATM. Search string (verbatim): ("Penile Prosthesis"[mh] OR "Penile Implantation"[mh] OR "penile prosthesis"[tiab] OR "penile implant"[tiab] OR "inflatable penile prosthesis"[tiab] OR "artificial penis"[tiab]) AND ("Operating Rooms"[mh] OR "Ventilation"[mh] OR "Air Conditioning"[mh] OR "Environment, Controlled"[mh] OR "laminar airflow"[tiab] OR "laminar flow"[tiab] OR "unidirectional airflow"[tiab] OR UDF[tiab] OR ventilation[tiab] OR HEPA[tiab] OR ultraclean[tiab] OR "air changes"[tiab] OR ACH[tiab] OR "air distribution"[tiab] OR "positive pressure"[tiab] OR "operating room"[tiab] OR "operating theatre"[tiab] OR "operating theater"[tiab]) AND ("Surgical Wound Infection"[mh] OR "Prosthesis-Related Infections"[mh] OR "surgical site infection"[tiab] OR infect*[tiab] OR explant*[tiab] OR revis*[tiab] OR reoperat*[tiab]).

*Embase (via Ovid)* - searched 27-28 September 2025. (Ovid syntax: .mp. multipurpose; exp explode.) Command-line (verbatim): 1 penile implant.mp. or exp penis prosthesis/ 2 Inflatable penile prosthesis.mp. or exp inflatable penis prosthesis/ 3 artificial penis.mp. or exp penis prosthesis/ 4 laminar airflow.mp. or exp laminar airflow/ 5 airflow/ or unidirectional airflow.mp. or air conditioning/ 6 exp operating room/ or operating theatre.mp. 7 surgical site infection.mp. or exp surgical infection/ 8 Prosthesis Removal.mp. or exp hardware removal/ 9 1 or 2 or 3 10 exp air quality/ or air distribution.mp. 11 exp medical device contamination/ or exp contamination/ 12 exp infection/ or exp prosthesis infection/ 13 4 or 5 or 6 or 10 14 7 or 8 or 11 or 12 15 9 and 13 and 14

*Cochrane Library - CENTRAL (Wiley Search Manager)* - searched 28 September 2025. Field labels :ti,ab,kw; proximity NEAR/n. Strategy (verbatim): #1 ("penile prosthesis" OR "penile implant" OR "inflatable penile prosthesis" OR "artificial penis"):ti,ab,kw #2 ("laminar airflow" OR "laminar flow" OR "unidirectional airflow" OR UDF OR ventilation OR HEPA OR ultraclean OR "air changes" OR ACH OR "air distribution" OR "positive pressure" OR "operating room" OR "operating theatre" OR "operating theater" OR (operating NEAR/1 room)):ti,ab,kw #3 (infection OR "surgical site infection" OR SSI OR explant* OR revis* OR reoperat* OR contaminat* OR coloniz* OR biofilm*):ti,ab,kw #4 #1 AND #2 AND #3 → 0 #5 MeSH descriptor: [Penile Prosthesis] explode all trees #6 MeSH descriptor: [Penile Implantation] explode all trees #7 MeSH descriptor: [Environment, Controlled] explode all trees #8 MeSH descriptor: [Operating Rooms] explode all trees #9 MeSH descriptor: [Surgical Wound Infection] explode all trees #10 MeSH descriptor: [Ventilation] explode all trees #11 MeSH descriptor: [Air Conditioning] explode all trees #12 MeSH descriptor: [Prosthesis-Related Infections] explode all trees #13 #5 OR #6 #14 #7 OR #8 OR #10 OR #11 #15 #9 OR #12 #16 #13 AND #14 AND #15 → 28 #17 #4 OR #16 → 28

*CINAHL (via EBSCOhost; Advanced Search) *- searched 28 September 2025. Field codes TI/AB; proximity N2. Strategy (verbatim): (TI("penile prosthesis" OR "penile prostheses" OR "penile implant*" OR "inflatable penile prosthesis" OR "artificial penis") OR AB("penile prosthesis" OR "penile prostheses" OR "penile implant*" OR "inflatable penile prosthesis" OR "artificial penis")) AND (TI("laminar flow" OR "laminar air flow" OR "unidirectional airflow" OR "unidirectional air flow" OR ultraclean OR UCV OR UDAF OR canopy OR ventilation OR "air change*" OR ACH OR "air distribution" OR "positive pressure" OR (operating N2 (room OR theatre OR theater))) OR AB("laminar flow" OR "laminar air flow" OR "unidirectional airflow" OR "unidirectional air flow" OR ultraclean OR UCV OR UDAF OR canopy OR ventilation OR "air change*" OR ACH OR "air distribution" OR "positive pressure" OR (operating N2 (room OR theatre OR theater)))) AND (TI(infect* OR "surgical site infection" OR SSI OR explant* OR revision* OR reoperat* OR contaminat* OR coloniz* OR colonis* OR biofilm*) OR AB(infect* OR "surgical site infection" OR SSI OR explant* OR revision* OR reoperat* OR contaminat* OR coloniz* OR colonis* OR biofilm*)) 

*Dimensions (Title and Abstract index) *- searched 27-28 September 2025. Boolean/phrases supported in basic search; proximity/wildcards require Advanced/DSL. Strategy (verbatim): ("penile prosthesis" OR "penile prostheses" OR "penile implant" OR "penile implants" OR "inflatable penile prosthesis" OR "inflatable penile prostheses" OR "artificial penis") AND ("laminar flow" OR "laminar airflow" OR "unidirectional airflow" OR "unidirectional air flow" OR UDF OR ventilation OR HEPA OR ultraclean OR "ultraclean air" OR "air change" OR "air changes" OR ACH OR "air distribution" OR "positive pressure" OR "operating room" OR "operating theatre" OR "operating theater" OR "ceiling-mounted canopy") AND (infection OR infections OR "surgical site infection" OR SSI OR explant OR explants OR "device removal" OR revision OR revisions OR reoperation OR reoperations OR "re-operation" OR "re-operations" OR contamination OR contaminations OR colonization OR colonisation OR biofilm OR biofilms)

*ClinicalTrials.gov (Advanced Search; "Other terms") *- searched 28 September 2025. Query (verbatim): ("penile prosthesis" OR "penile implant" OR "inflatable penile prosthesis" OR "artificial penis") AND ("laminar airflow" OR "laminar flow" OR "unidirectional airflow" OR "unidirectional flow" OR ultraclean OR UCV OR UDAF OR ventilation OR HEPA OR "air changes" OR ACH OR "operating room" OR "operating theatre" OR "operating theater") AND (infection OR infections OR "surgical site infection" OR SSI OR explant OR explantation OR revision OR revisions OR reoperation OR reoperations OR "device removal" OR contamination OR colonization OR colonisation OR biofilm OR biofilms) Filters/limits: none (registry interface). Yield: 0 eligible trials (no comparative PP ceiling-mounted LAF/UCV outcomes identified).

*WHO ICTRP Search Portal (trialsearch.who.int) *- searched 28 September 2025. Query (verbatim): ("penile prosthesis" OR "penile implant" OR "inflatable penile prosthesis") AND (laminar OR "laminar airflow" OR ultraclean OR ventilation OR HEPA OR "unidirectional airflow" OR UCV OR UDAF) AND (infection OR "surgical site infection" OR SSI OR explant* OR revision* OR reoperat*) Filters/limits: none (portal interface). Yield: 0 eligible trials.

*ISRCTN *- searched 28 September 2025. Query (verbatim): ("penile prosthesis" OR "penile implant" OR "inflatable penile prosthesis") AND (laminar OR "laminar airflow" OR ultraclean OR ventilation OR HEPA OR "unidirectional airflow" OR UCV OR UDAF) AND (infection OR "surgical site infection" OR explant* OR revision* OR reoperat*) Filters/limits: none (registry interface). Yield: 0 eligible trials.

Websites (guideline portals)

*AUA (auanet.org) *- site-restricted web search - run date: 5 October 2025. Query (verbatim): site:auanet.org ("penile prosthesis" OR "penile implant" OR "inflatable penile prosthesis") (laminar OR "laminar airflow" OR ultraclean OR UCV OR UDAF OR ventilation OR HEPA OR "operating room" OR "operating theatre"). Search engine used: Google (site: operator). Yield: 0 eligible comparative PP LAF/UCV studies (context only).

*EAU (uroweb.org) *- site-restricted web search - run date: 5 October 2025. Query (verbatim): site:uroweb.org ("penile prosthesis" OR "penile implant" OR "inflatable penile prosthesis") (laminar OR "laminar airflow" OR ultraclean OR UCV OR UDAF OR ventilation OR HEPA OR "operating room" OR "operating theatre"). Search engine used: Google (site: operator). Yield: 0 eligible comparative PP LAF/UCV studies (context only).

Citation chasing

Backward (reference-list) and forward (cited-by) citation searching was conducted for the full-text reports assessed (Mulcahy et al., 2014; and the HUAIRS abstract/manufacturer material). Forward citation searching used Web of Science and Google Scholar ("cited by"), and backward citation searching involved screening the reference lists of the seed reports. Run: 27-28 September 2025. Yield: 1 additional record (counted under "Other sources" in PRISMA Identification); no additional eligible comparative PP LAF/UCV studies were identified.

Appendix 2: Full-text reports assessed for eligibility and excluded (primary reason)

**Table 5:** Full-text reports assessed for eligibility and excluded (primary reason) Exposure definition: LAF/UCV denotes a ceiling canopy-defined unidirectional (laminar/ultraclean) flow field as specified in ventilation standards/guidance. Portable HEPA/UV recirculators (e.g., HUAIRS) recirculate room air and are not LAF/UCV exposures; excluded a priori. LAF - laminar airflow; UCV - ultraclean ventilation; PP - penile prosthesis; OR - operating room; HEPA - high-efficiency particulate air

Study (citation)	Year	Report type	Field/procedure	Ventilation exposure reported?	Primary reason for exclusion (single-coded)	Notes	DOI / URL	Accessed
Mulcahy JJ, et al. Current management of penile implant infections, device reliability, and optimizing cosmetic outcome [21]	2014	Full text (review)	Penile prosthesis (PP)	No (not a comparative ventilation study)	Wrong design (secondary research)	Narrative review; no ventilation comparison; no infection outcomes stratified by LAF/UCV vs conventional.	10.1007/s11934-014-0413-6	October 5, 2025
Airborne Bacteria in the Operating Room Can Be Reduced by HEPA/Ultraviolet Air Recirculation System (HUAIRS) (Surgical Infection Society Annual Meeting abstract) [22]	2017	Conference abstract/ manufacturer material	OR environmental control (non-canopy portable recirculator)	Not LAF/UCV canopy	Wrong exposure (not LAF/UCV canopy); insufficient data	Device-level particle/CFU reductions in room/bench settings; no comparative PP infection outcomes reported.	SIS abstract; Aerobiotix “Evidence in Brief” (Illuvia HUAIRS). https://aerobiotix.com/wp-content/uploads/2021/06/Illuvia-HUAIRS-Evidence-Pocketbook-PRINT.pdf	October 5, 2025

Appendix 3: PRISMA 2020 counts table

**Table 6:** PRISMA 2020 counts table LAF/UCV - laminar airflow/ultraclean ventilation; HEPA/UV - high-efficiency particulate air/ultraviolet; ICTRP - International Clinical Trials Registry Platform; PP - penile prosthesis

PRISMA node	Count	Notes
Records identified from databases (pre-deduplication)	98	MEDLINE 9; Embase 34; CENTRAL 27; CINAHL 1; Dimensions 27
Records identified from registers	0	ClinicalTrials.gov 0; WHO ICTRP 0; ISRCTN 0
Records identified from websites/organisations/citation searching	1	JSM 2023 supplement abstract (portable HEPA/UV)
Duplicates removed before screening	26	8 EndNote; 18 Rayyan (PRISMA groups all duplicates before screening)
Marked ineligible by automation tools	0	-
Removed for other reasons	0	-
Records screened (titles/abstracts)	72	Databases 98 - duplicates 26 = 72; the 1 record from other methods bypassed title/abstract screening and is counted at “Reports assessed for eligibility".
Reports assessed for eligibility (full-text/other reports)	2	Databases = 1 (Mulcahy 2014, review); other sources = 1 (HUAIRS website/abstract)
Full-text/other reports excluded (with primary reason)	2	Wrong design (secondary research); Wrong exposure (not LAF/UCV canopy)
Studies included in qualitative synthesis	0	No eligible comparative PP studies by ventilation mode
Studies included in quantitative synthesis (meta-analysis)	0	No meta-analysis performed

Appendix 4: Key LAF/UCV perturbations, mechanistic airflow effects, and practical mitigations

**Table 7:** Laminar airflow and ultraclean ventilation: common operating-room perturbations, mechanistic airflow effects, and practical mitigations This conceptual table summarises practical factors that can degrade intended unidirectional (laminar) flow over the sterile field (e.g., door openings/traffic, staff/equipment movement, thermal plumes, clutter, and maintenance issues) and the resulting airflow phenomena reported in operating-room ventilation and computational studies (entrainment, wakes, recirculation, buoyancy-driven updrafts, and dead zones), together with pragmatic mitigations (traffic/door discipline, layout/equipment positioning outside the downflow, management of heat sources and lights, verification of supply alignment/velocity/coverage, and routine filtration/maintenance). ACH - air changes per hour; CFD - computational fluid dynamics; HEPA - high-efficiency particulate air; OR - operating room

Perturbations	Mechanistic effects	Practical mitigations
Door openings/traffic	Entrainment; mixing/turbulence	Door discipline; minimise traffic; use an anteroom where available
Staff movement/equipment movement	Shear/wakes; entrainment	No-cross zones; standardised traffic patterns; minimise non-essential movement
Thermal misalignment/undersized footprint	Buoyant updrafts opposing downflow; recirculation	Manage heat sources (warming devices); adjust light angle/intensity; verify canopy alignment/coverage
Low supply velocity or intra-case ACH turn-down	Dead zones; reduced clearance	Verify supply velocity; maintain ACH; extend diffuser footprint where feasible
Equipment placed in the airstream	Flow separation/shadowing; recirculation	Stage equipment outside downflow; reposition equipment/cables; park booms outside the airstream
Crowded layouts/high occupancy	Recirculation; dead zones	Limit occupancy; declutter; keep the field unobstructed
Maintenance gaps	Reduced filtration/flow performance	Scheduled HEPA filter changes; routine maintenance/commissioning checks

Appendix 5:  The distribution and overall direction of clinical findings across specialties 

Appendix 6: Key bias pathways and design safeguards for LAF study design

**Table 8:** Bias and confounding - study-design toolbox for evaluating laminar airflow (LAF) in penile prosthesis surgery Illustrative framework for planning and interpreting non-randomised evaluations of LAF in penile prosthesis surgery. Threats include confounding by indication (e.g., higher-risk/revision cases preferentially scheduled), secular trends around installation periods/behavioural shifts, and in-use breaks in unidirectionality from traffic and door openings. Design choices include preregistration to limit analytic flexibility, defining an a priori adjustment set (revision status, diabetes control/HbA1c, operative duration, device calendar time, hospital fixed effects), and propensity-score methods (matching or stabilised inverse-probability weighting). Diagnostics/robustness include prespecified sensitivity analyses (negative-control outcomes/falsification tests and the E-value for unmeasured confounding). Items are illustrative, not exhaustive, and should be implemented within a prespecified analysis plan. HbA1c - glycated haemoglobin; LAF - laminar airflow

Threats	Design choices	Diagnostics/robustness
Confounding by indication (higher-risk/revision cases may cluster).	Preregister analysis; limit analytic flexibility.	Prespecify sensitivity analyses.
Secular trends (installation period; behavioural shifts over time).	Prespecify adjustment set (revision status; diabetes/HbA1c; operative duration; calendar time; hospital fixed effects).	Negative-control outcome(s) and falsification tests.
Loss of unidirectionality in use (traffic/door openings degrade laminarity).	Propensity-score methods (matching or stabilised inverse-probability weighting).	E-value for unmeasured confounding.

Appendix 7: The pragmatic research agenda for LAF

Appendix 8: Standard operating-theatre ventilation requirements

**Table 9:** Standard operating-room ventilation requirements and how they reduce infection risk This table summarises commonly specified operating-room ventilation parameters (pressure relationship, total and outdoor air-change rates, filtration, supply/return configuration, temperature/relative humidity set-points, and door policy) and the infection-control rationale (airborne bioburden dilution and aerosol clearance; directional clean-air sweep over the sterile field; reduced contaminant ingress via positive pressure; particulate capture by filtration; and reduced deposition/resuspension when traffic and turbulence are minimised). Parameters align with widely used guidance, including ANSI/ASHRAE/ASHE Standard 170 and NHS HTM 03-01, with door-closure/limited entry supported by CDC environmental infection-control recommendations. Minimum targets can vary by jurisdiction and local specification (for example, CDC guidance lists ≥15 total ACH and ≥3 outdoor ACH for operating rooms, whereas Standard 170 specifies higher minimum requirements). ACH - air changes per hour; CFU - colony-forming units; HEPA - high-efficiency particulate air; MERV - minimum efficiency reporting value; OR - operating room; RH - relative humidity

Requirements	How it helps reduce infection
Positive pressure to adjacent spaces (maintained when occupied).	Pressurisation barrier: reduces ingress of contaminated air from adjacent areas.
Ventilation rate: ≥20 air changes per hour (ACH) total; ≥4 ACH outdoor (fresh) air.	Dilution + clearance: lowers airborne microbial load and speeds aerosol removal.
Filtration: two-stage system; final filter ≥MERV-14 (MERV-16 typical); terminal HEPA where ultraclean performance is required.	Filtration capture: removes particulates/microorganisms and reduces downstream contamination.
Airflow pattern: ceiling supply array with unidirectional downflow over the sterile field; low-level returns at the periphery.	Directional sweep: delivers cleaner air to the field and promotes contaminant removal away from the incision.
Environmental set-points: 20–24 °C; relative humidity 20–60%.	Supports stable airflow performance and avoids extremes that can worsen condensation/static/dust behaviour.
Door/traffic control: doors closed; minimise door openings; system runs continuously except during maintenance.	Less turbulence + fewer pressure disturbances: reduces contamination entry and dust resuspension from traffic.
